# A six lipid metabolism related gene signature for predicting the prognosis of hepatocellular carcinoma

**DOI:** 10.1038/s41598-022-25356-2

**Published:** 2022-12-01

**Authors:** Kequan Xu, Peng Xia, Pan Liu, Xiao Zhang

**Affiliations:** 1 Department of Hepatobiliary and Pancreatic Surgery, Zhongnan Hospital of Wuhan University, Wuhan, 430071 People’s Republic of China; 2 Department of Hepatobiliary and Pancreatic Surgery, People’s Hospital of Zhengzhou University, Zhengzhou, People’s Republic of China

**Keywords:** Cancer, Cancer metabolism, Cancer microenvironment, Oncogenes

## Abstract

Globally, hepatocellular carcinoma (HCC) is one of the most lethal malignant tumors. Studies have shown that alterations in the tumor immune microenvironment (TIME) play a significant role in the pathogenesis and progression of HCC, and notably, lipid metabolism has been shown to regulate TIME. Therefore, in predicting the prognosis and efficacy of immunotherapy in patients with HCC, lipid metabolism-related prognostic factors are highly relevant. mRNA expression data of HCC were obtained from the Cancer Genome Atlas (TCGA), International Cancer Genome Consortium (ICGC), and gene expression omnibus (GEO) databases. and lipid metabolism-related genes were also obtained from the GSEA databases. Least absolute shrinkage and selection operator regression analysis, univariate and multivariate Cox proportional hazards analysis were used to explore lipid metabolism-related prognostic genes and further construct a prognostic signature in the training set, ICGC and GSE54236 were used to validate the accuracy of the signature. qRT-PCR was used to detect the mRNA levels of lipid metabolism-related prognostic genes in HCC tissues and their paired adjacent tissues. Nile red staining was used to demonstrate lipid content in HCC tissues. Immunofluores-cence and ELISA were used to detect immune cells and immune responses in HCC tissues and serum. Six lipid metabolism-related genes (ADH1C, APEX1, ME1, S100A10, ACACA and CYP2C9) were identified as independent prognostic factors, which were used for risk model construction for HCC patients. The areas under the 1-, 2-, and 3-year ROC curves for the TCGA cohort were 0.758, 0.701 and 0.671, respectively. Compared with paired paracancerous tissues, qRT-PCR revealed that APEX1, ME1, S100A10 and ACACA were up-regulated in HCC tissues, whereas ADH1C and CYP2C9 were down-regulated in HCC tissues. Nile red staining indicated that this study showed that both the HCC tissue and serum of patients in the high-risk group exhibited lipid accumulation. Our identified prognostic model comprising six lipid metabolism-related genes could provide survival prediction. Moreover, HCC drug therapy target selection and molecular marker research can be guided by our predictive model.

## Introduction

In terms of morbidity and mortality, hepatocellular carcinoma (HCC) is one of the most lethal solid tumors^1^. Although there have been many targeted therapies for HCC in recent years, there are still many HCC patients with unsatisfactory prognosis, postoperative recurrence and metastasis rates^2–4^. For the early diagnosis and precise treatment of HCC, there is an urgent need to identify effective targets or biomarkers.

Recent studies have shown that metabolic reprogramming is regarded as a core feature of cancer and that lipid metabolism reprogramming plays a pivotal role in the development of HCC. A dysregulation of lipid metabolism, especially fatty acid metabolism, in which oncogenic signaling pathways are abnormally activated, alters the expression and activity of lipid metabolizing enzymes. This is becoming increasingly recognized as a metabolic rewiring of tumor cells. The accumulation of lipids increases tumor cell viability and exacerbates HCC progression^5,6^. Numerous studies have linked lipid metabolism to the immune system and immune-related functions^7^. Studies have shown that high levels of E-FABP-expressing tumor-associated macrophages (TAMs) in HCC could recruit natural killer (NK) cells and drive anti-cancer immune responses^8^. Another study reported that tumor-derived factors trigger lipid peroxidation in tumor-associated DCs (TADCs), thereby activating inositol-requiring protein 1α (IRE-1α) and its target X-box-binding protein 1 (XBP1) -mediated endoplasmic reticulum stress response. In addition, XBP1 activation can lead to diminished lipid accumulation and antigen presentation, resulting in a reduced ability for tumors to grow^9^. Taken together, the lipid metabolism plays a key role in tumorigenesis and tumor progression, and its potential as an immunotherapy target is significant.

The study was based on the close relationship between lipid metabolism and immunity. In this study, we constructed a prognostic model based on six genes related to lipid metabolism (ADH1C, APEX1, ME1, S100A10) using least absolute shrinkage and selection operator regression analysis (LASSO). Three databases (TCGA, ICGC, and GSE54236) were used to validate its predictive ability using survival analysis and receiver operating characteristic (ROC) curves. We investigated the relationship between risk score and immune infiltration using immune infiltration analysis. We validated the prognostic value, lipid metabolism and immune function of the prognostic model in HCC with specimens from People’s Hospital of Zhengzhou University. The understanding of dysregulated lipid metabolism in HCC could suggest new treatment strategies for HCC through modulation of the tumor microenvironment (TME) by reprogramming cellular lipid metabolism. In addition, HCC patients can benefit from our findings regarding tumor immune microenvironment (TIME) characterization, which can help in clinical management and decision-making.

## Materials and methods

### Data collection

The Cancer Genome Atlas (TCGA) database was used to download mRNA sequencing data for 50 normal samples and 374 liver cancer samples. Moreover, we downloaded mRNA sequencing data from the International Cancer Genome Consortium (ICGC) database and GSE54236 for 240 and 81 HCC patients, respectively. The analysis included 370 patients from the TCGA database, 232 patients from the ICGC database, and 81 patients from GSE54236 after excluding patients with incomplete data. A total of 135 patients without complete clinical information were excluded from the TCGA analysis of clinical relevance. From GSEA databases, 8 pathways and 323 genes related to lipid metabolism were identified. All websites used are shown in table S1.

### Differentially expressed genes and prognosis-related genes identified

A differentially expressed gene analysis was performed using the “limma” R package. Screening conditions were log2-fold change (logFC) > 1 and false discovery rate (FDR) < 0.05. The prognosis of genes associated with lipid metabolism was identified using the “survival” R package. Transcription factors in HCC were obtained from the Cistrome database, and Cytoscape was used to generate transcription factor regulatory networks for overlapping genes (*p* > 0.001, cor > 0.5).

Prognostic models (Risk score = (− 0.000934507 × ADH1C expression) + (0.010025652 × APEX1 expression) + (0.029611698 × ME1 expression) + (0.001505404 × S100A10 expression) + (0.067765234 × ACACA expression) + (− 0.001361529 × CYP2C9 expression).) were constructed using LASSO regression and multivariate Cox regression based on TCGA data. After the model is validated, we perform survival analyses, ROC curve analyses, univariate and multivariate analysis to check its predictive power from ICGC and GSE54236 data.

### Analysis of immune cell infiltration and gene set enrichment in risk-related genes

In TCGA data analysis, 94 differential genes related to lipid metabolism were analyzed using GO functional enrichment analysis. To perform enrichment analysis, we used the the “clusterProfiler” and “org.Hs.eg.db” R packages with *P*-values of 0.05 and q-values of 0.05 as thresholds. From TCGA, we perform enrichment analysis of genes in high and low risk groups. In addition, for each sample, we calculated the relationship between the risk score and immune-related score using the Single-Sample Gene Set Enrichment Analysis (ssGSEA) function in the “GSVA” R package.

### Tissue sample

A total of 40 pairs of HCC tissues and matched nontumour tissues were collected from HCC patients at People's Hospital of Zhengzhou University (Henan, China), with written informed consent. Preoperative chemotherapy or radiotherapy was administered to each one of the patients in this study. The Ethics Committee of People's Hospital of Zhengzhou University (2022NO.33) approved this study in accordance with the Declaration of Helsinki.

### RNA extraction and real-time quantitative PCR (qRT-PCR)

Total RNA was extracted with TRIzol (T9108, Takara, Dalian, China) and reverse transcribed using an enzyme kit. Then, 2 × ChamQ Universal SYBR qPCR Master Mix (Q711-02, Vazyme, Nanjing, China) was used for qRT-PCR. The sequences of all PCR primers used are as table S2 showed.

### Triglyceride assays

A triglyceride assay kit (E1013-105, Applygen Technologies Inc, Beijing, China) was used to assess intracellular and intratumoural triglyceride contents according to the manufacturer's instructions.

### Nile red staining

The sufficiently homogenized tissues were fixed in 6-well plates with 4% paraformaldehyde solution, washed with PBS and stained with Nile Red solution (7385-67-3, Solarbio, Beijing, China) in the dark. In IF microscopy, images were acquired after the samples were washed twice with PBS and stained with DAPI.

### Immunofluorescence microscopy

Samples were blocked with 1% bovine serum albumin in PBS for 30 min, and then incubated with CD56 antibody (A7913, ABclonal, Wuhan, China). Slides were observed with a fluorescence microscope using DAPI (C1002, Beyotime Biotechnology) as secondary antibody.

### Serum cytokine analysis

Serum IFNα levels were examined using IFN alpha Human ELISA Kit (BMS216, Thermo Fisher, Shanghai, China). Serum IFNβ levels were examined using IFN beta Human ELISA Kit (414,101, Thermo Fisher, Shanghai, China). Serum IFNγ levels were examined using IFN gamma Human ProQuantum Immunoassay Kit (A35576, Thermo Fisher, Shanghai, China).

### Statistical analysis

Software used for statistical analysis was GraphPad Prism 8.0 (GraphPad Software, San Diego, California, USA) and SPSS 24.0 (SPSS Inc., Chicago, Illinois, USA). It is assumed that each experiment included at least three independent experiments. Continuous variables are expressed as mean ± standard deviation (SD). To compare continuous variables, unpaired Student's t-tests were used. Student's t-test or one-way ANOVA was used to assess differences between experimental groups. Cox regression or Kaplan–Meier analysis were used to determine survival time between groups. A significance level of 0.05 was determined based on two-sided *P*-values. *P < 0.05; ∗  ∗ *P* < 0.01; ***p < 0.001.

### Ethics approval and consent to participate

A total of 40 pairs of HCC tissues and matched nontumour tissues were collected from HCC patients at People's Hospital of Zhengzhou University (Henan, China), with written informed consent. Preoperative chemotherapy or radiotherapy was administered to each one of the patients in this study. The Ethics Committee of People's Hospital of Zhengzhou University (2022NO.33) approved this study in accordance with the Declaration of Helsinki.

## Results

### Screening of prognostic genes related to lipid metabolism

**Figure 1:**
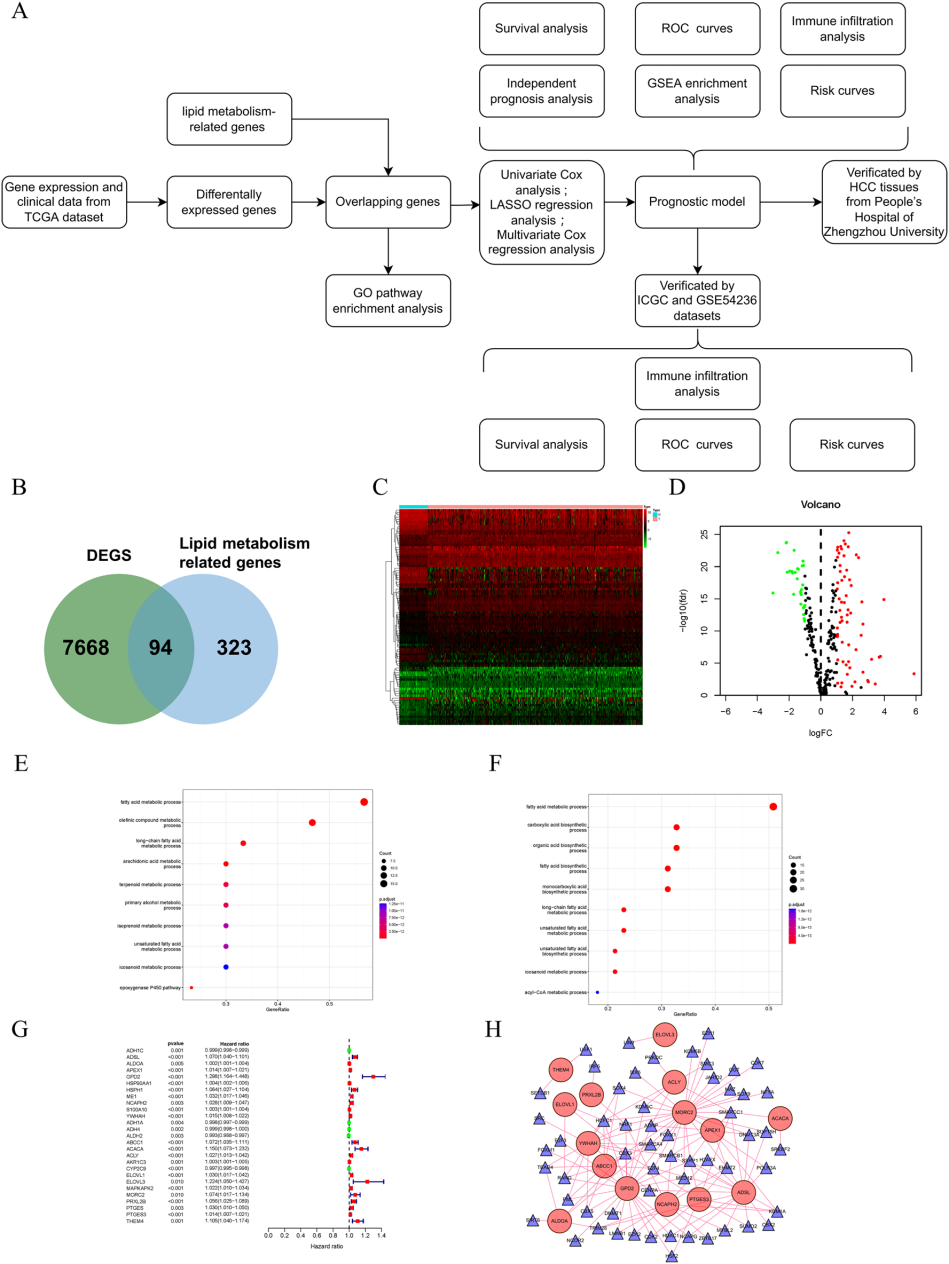
Differentially expressed and lipid metabolism related and prognosis-related genes. (**A**) Flowchart of the study. (**B**) Venn diagram of 7668 differential expressed genes and 323 genes related to lipid metabolism. (**C**) Heat map of overlapping genes (using “pheatmap” R package, https://cran.r-project.org/bin/windows/base/old/4.1.3/). (**D**) Volcano map of overlapping genes. (**E**,**F**) GO pathway enrichment analysis of TCGA cohorts. (**G**) Hazard ratios of prognosis-related genes. (**H**) Transcription factor regulatory network. Red, positive regulation; green, negtitive regulation.

AS Fig. 1A showed, the TCGA database was used to obtain 374 HCC samples and 50 normal samples. The sequencing data itself contained 60,483 sets of RNA expression data, and 7668 different expressed genes (DEGs) were analyzed by the “limma” R package (log foldchange > 2, *p* < 0.01). Next, 94 differential genes related to lipid metabolism in HCC were obtained by intersecting with the lipid metabolism-related genes obtained from GESA databases, including 63 up-regulated and 30 down-regulated lipid metabolism-related genes (Fig. 1B). Results are presented in the form of heatmaps and volcano plots (Fig. 1C,D). Next, we performed GO enrichment analyses to further understand the functions of these genes and to confirm their roles in lipid metabolism. Our results showed that multiple GO terms related to lipid metabolism were found in biological processes, and both up-regulated and down-regulated genes were enriched in “fatty acid metabolic process” (Fig. 1E,F). Next, we identified a total of 27 lipid metabolism-related differential genes using univariate Cox analysis (Fig. 1G). Since transcription factors play an important role in lipid metabolism, we generated a transcription factor regulatory network to explore possible transcription factors regulating lipid metabolism genes associated with prognosis (*p* > 0.001, cor > 0.5). Gene regulatory relationships are represented by lines, positive correlations by red, and all transcription factors in this study are positive regulators. And most transcription factors regulate MORC2 and GPD2 (Fig. 1H).

### Construction of a prognostic model

**Figure 2:**
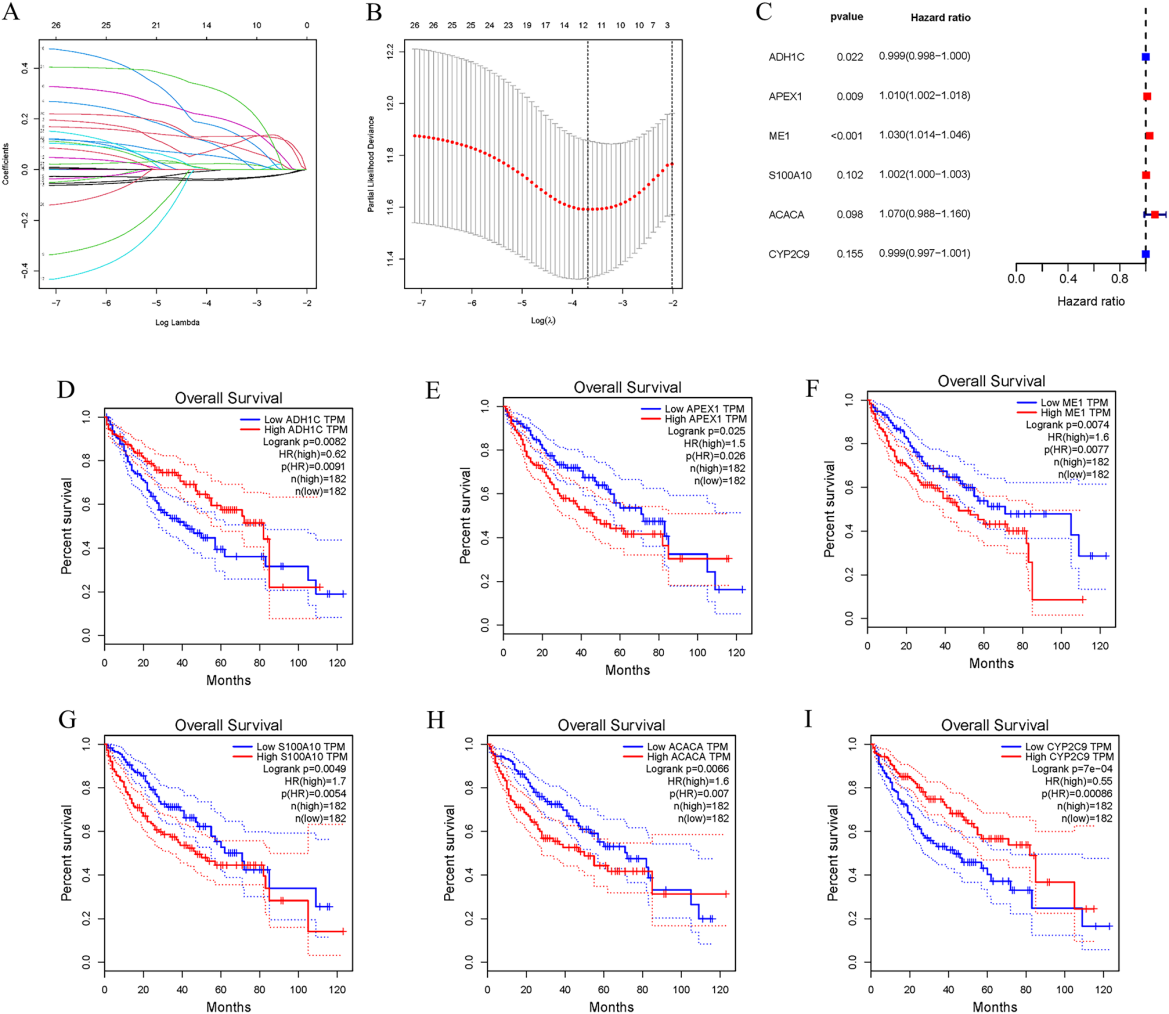
Construction of a prognostic model. (**A**) Plots for LASSO expression coefficients of 27 lipid metabolism related genes. (**B**) Cross-validation plot for the penalty term. (**C**) Relationship between four glycolysis-related lncRNAs and prognosis of HCC patients. (**D**–**I**) Survival curves of patients with differential expression of six genes in TCGA.

We next performed LASSO regression analysis on 27 genes to rule out overfitting and identified 13 genes related to lipid metabolism (Fig. 2A,B). In HCC samples, six lipid metabolism-related genes were found to be associated with prognosis using multivariate Cox regression analysis (Fig. 2C). Among them, APEX1, ME1, S100A10 and ACACA were found to be negative prognostic factors (Hazard ratio, HR > 1) and were upregulated in HCC samples (Figure S1). On the other hand, ADH1C and CYP2C9, which are protective factors (HR < 1), were found to have reduced expression in HCC samples (Figure S1). Furthermore, HCC patients with higher APEX1, ME1, S100A10 and ACACA expression or lower ADH1C and CYP2C9(*p* < 0.05) expression had poorer prognosis (Fig. 2D–I). Based on the expression of lipid metabolism-related genes and multivariate Cox regression coefficient, the prognostic risk score for each sample was calculated as follows:Risk score = (− 0.000934507 × ADH1C expression) + (0.010025652 × APEX1 expression) + (0.029611698 × ME1 expression) + (0.001505404 × S100A10 expression) + (0.067765234 × ACACA expression) + (− 0.001361529 × CYP2C9 expression).

### Risk score significantly correlated with patient prognosis and HCC pathological features

**Figure 3:**
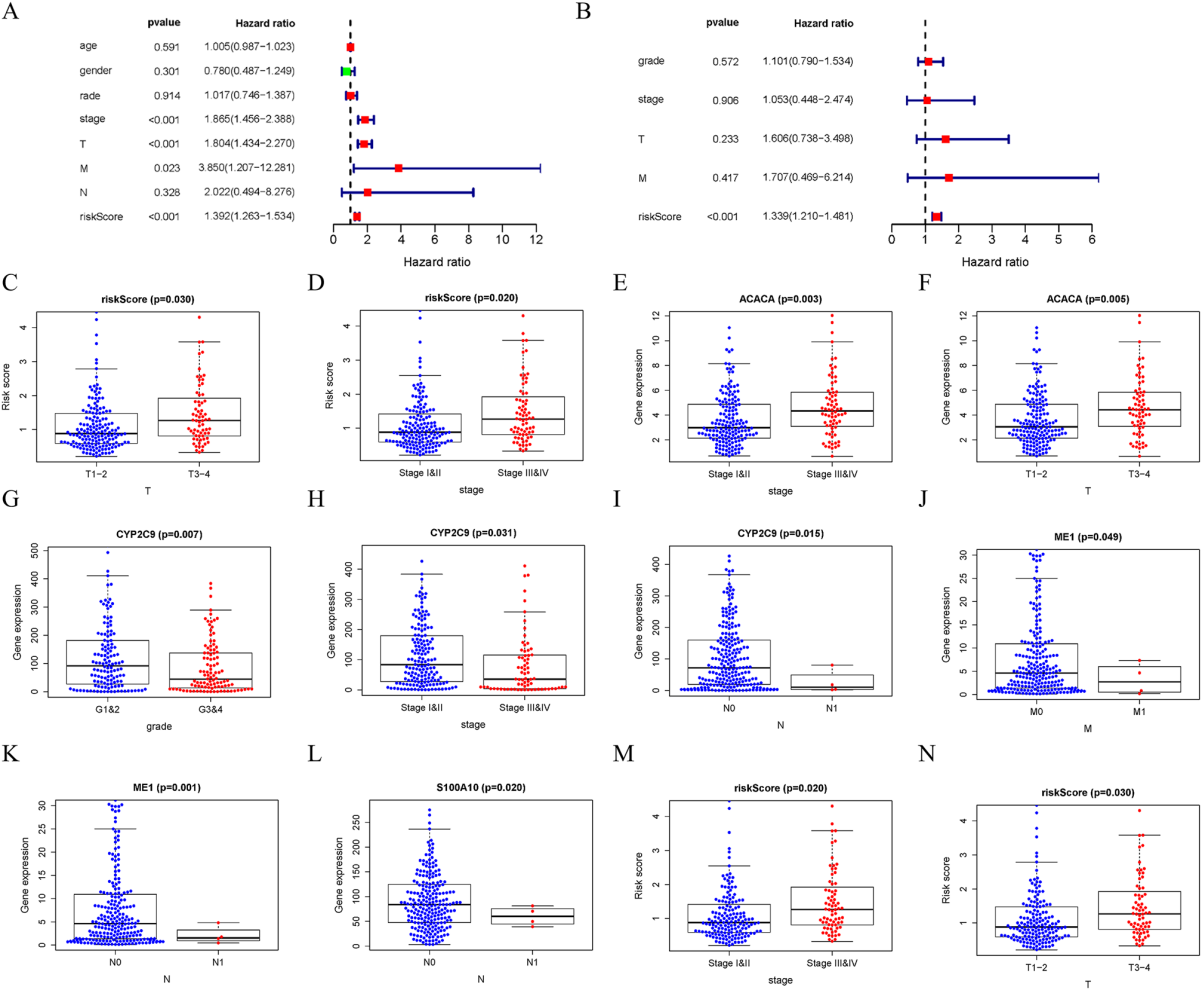
Risk score significantly correlated with patient prognosis and HCC pathological features. (**A**) Univariate Cox regression analysis of TCGA cohort. (**B**) Multivariate Cox regression analysis of TCGA cohort. (**C**–**N**) Relationship between risk score and gene expression and clinicopathological features of HCC.

For each patient, a prognostic risk score was calculated. After performing multivariate and univariate Cox regressions on patients' risk scores, we performed independent prognostic analysis. Multivariate prognostic analysis showed that disease stage, T stage, M stage and risk score were independent prognostic factors (Fig. 3A). Multivariate prognostic analysis revealed that only risk score (*p* < 0.001) was an independent prognostic factor, and risk score had a high hazard ratio (Fig. 3B). In addition, correlation analysis with clinical case characteristics showed that patients' risk scores were significantly associated with disease stage and T stage (*p* < 0.05), while gene expression within the model was also significantly associated with different disease pathological characteristics (*p* < 0.05) (Fig. 3C–N).

### Validation of prognostic risk scores

**Figure 4:**
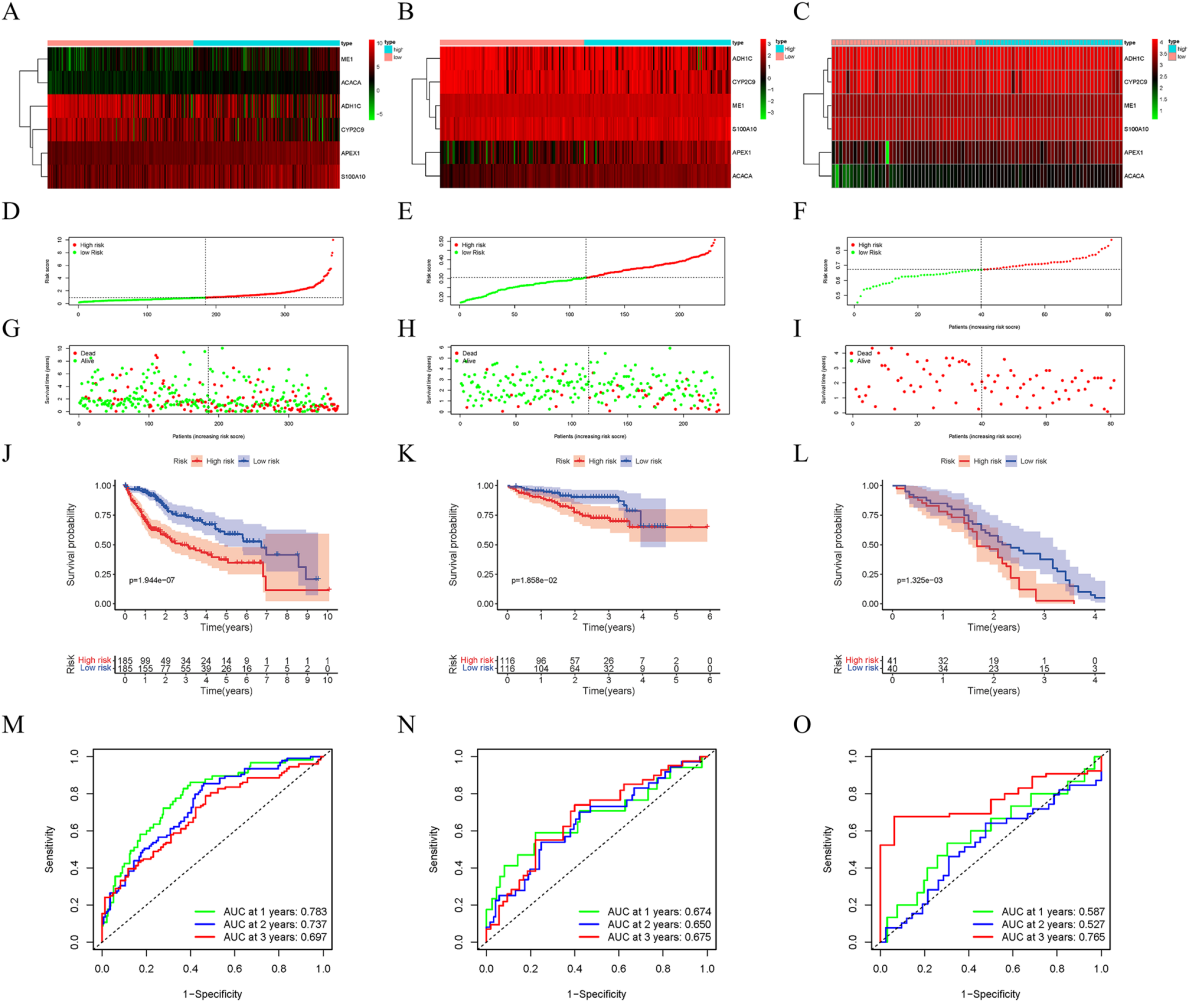
Verification of the prognostic models. Heat map (using “pheatmap” R package, https://cran.r-project.org/bin/windows/base/old/4.1.3/) of the expression of 6 lipid metabolism related genes in HCC samples in TCGA (**A**), ICGC (**B**) and GSE54236 (**C**). (**D**–**F**) Distribution of risk scores for HCC samples in above three datasets. (**G**–**I**) The relationship between survival time and status of HCC samples and risk score in above three datasets. (**J**–**L**) Kaplan–Meier survival curve of samples in high- and low-risk groups in above three datasets. (**M**–**O**) ROC curve of risk score in samples with HCC in above three datasets.

Figure 4A–C illustrates the heatmap distributions of six genes related to lipid metabolism in three databases (TCGA, ICGC, and GSE54236). A higher risk score resulted in fewer surviving samples and more dead samples. Compared to the high-risk group, the low-risk samples had significantly better prognoses (Fig. 4D–I). Compared with samples from the high-risk group, samples from the low-risk group had a significantly higher 5-year survival rate (Fig. 4J–L). Further, a high-precision prognostic scoring system was constructed using the lipid metabolism-related genes. The areas under the AUC curve at 1, 2, and 3 years were 0.783, 0.737 and 0.697 in the TCGA dataset, and 0.674, 0.650 and 0.675, respectively, in the ICGC dataset (Fig. 4M,N). In the GSE54236 dataset, the areas under the AUC curve at 1, 2, and 3 years were 0.587, 0.527, and 0.765, respectively (Fig. 4O). Our models in above three datasets showed great predictive potential for HCC patient prognosis.

### Risk score is closely related to tumor immune environment characteristics and immune infiltration in HCC

Further evidence for the potential role of risk scores in HCC TIME was obtained by examining the relationship between risk scores and immune-related scores using “ESTIMATE” R package. The ssGSEA method was used to analyze immune signatures, the CIBERSORT method to evaluate tumor-initiating cell subtypes, and 29 genes involved in immune checkpoint blockade.

**Figure 5:**
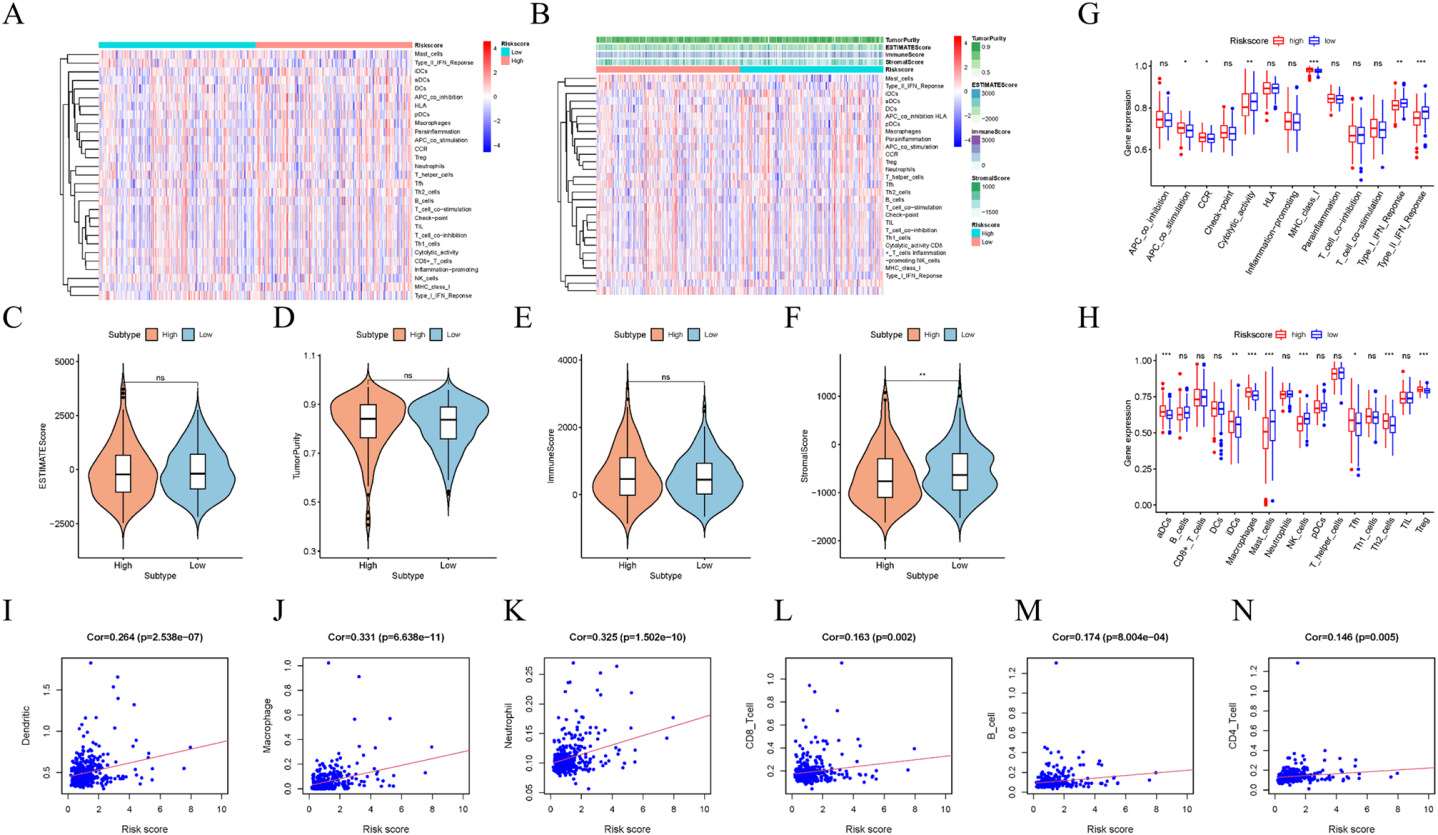
Risk score is closely related to tumor immune environment characteristics and immune infiltration in HCC. (**A**,**B**) Heat map (using “pheatmap” R package, https://cran.r-project.org/bin/windows/base/old/4.1.3/) distribution of risk score and various immune scores. (**C**–**F**) Comparison of estimate score, stromal score, immune score, and tumor purity between high-risk groups and low-risk groups. (**G**) The relationship between immune function and risk score in TCGA cohort. (**H**) The relationship between immune cell infiltration and risk score in the TCGA cohort. (**I**–**N**) Association between this signature and B cells, CD4 + T cells, CD8 + T cells, dendritic cells, macrophages cells and neutrophil cells.

Heatmap distributions of risk scores and various immune scores are shown (Fig. 5A,B). Compared with low-risk samples, high-risk samples had lower stroma scores (*p* < 0.01), but no significant differences were found in estimated scores, tumor purity, and immune scores (Fig. 5C–F). In the next step, we examined whether there were differences in immune signatures between low and high risk groups. From the ssGSEA results, we found that high-risk groups had significantly more infiltration levels of aDCs, iDCs, Macrophages, Tfh, Th2 cells, and Tregs, while the infiltration levels of Mast cells, NK cells were significantly decreased, and some immune characteristics (APC co stimulation, CCR, and MCHI classes) were significantly activated with increasing risk scores, and some immune features (Cyolytic activity, IFN classes I and II) were suppressed (Fig. 5G,H). In addition, through the validation of the ICGC and GSE54326 datasets, it was found that the three data all showed that the stroma scores decreased, NK cells decreased, and IFN classes I and II were inhibited in the high-risk group (Figure S2A–H, Figure S3A–H). Based on further correlation analysis, we found that the risk signature was significantly correlated with dendritic (r = 0.264; *p* = 2.538e-07), Microphage (r = 0.331; *p* = 6.638e-11), Neutrophil (r = 0.325; *p* = 1.502) e-10), infiltrating CD8 + T cells (r = 0.163; *p* = 0.002), infiltrating B cells (r = 0.174; *p* = 8.004e-4; Fig. 5I–N) and infiltrating CD4 + T cells (r = 0.146; *p* = 0.005). These results suggest that risk signatures based on lipid metabolism-related genes may help provide new insights into TIME signatures, immune responses, and immune infiltration in HCC.

### Expression of six lipid metabolism-related genes in hepatocellular carcinoma and their effect on prognosis

**Figure 6:**
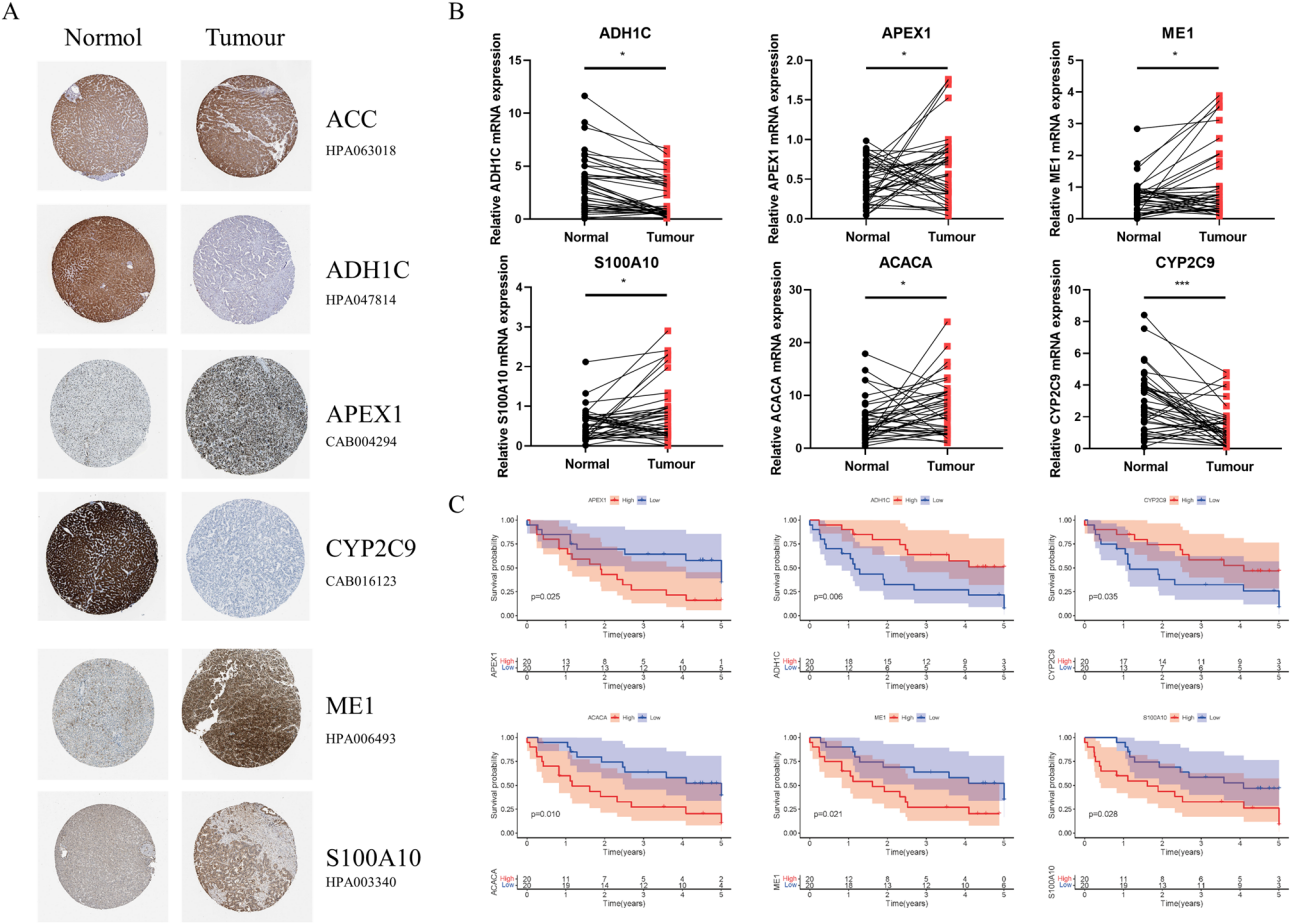
Expression of six lipid metabolism-related genes in hepatocellular carcinoma and their effect on prognosis. (**A**) Expression of risk-related genes in liver cancer tissues and adjacent normal tissues (HPA database). (**B**) 6 lipid metabolism related genes mRNA levels in tumor tissues and adjacent normal tissues (patients treated at People’s Hospital of Zhengzhou University). (**C**) Survival curve of patients with high and low risk scores (patients treated at People’s Hospital of Zhengzhou University).

IN HPA database, immunohistochemistry showed that the expressions of APEX1, ME1, S100A10 and ACACA in liver cancer tissues were significantly higher than those in paired normal liver tissues, while ADH1C, and CYP2C9 were expressed at low levels in tumor tissues compared with adjacent normal tissues (Fig. 6A). The qPCR detection of forty pairs of HCC tissues and their corresponding paracancerous tissues from People's Hospital of Zhengzhou University Hospital confirmed this diagnosis. (Fig. 6B). According to the follow-up and gene expression data of TCGA, high expression of APEX1, ME1, S100A10 and ACACA was closely associated with poor prognosis, while high expression of ADH1C, CYP2C9 improved the prognosis of HCC patients (*P* < 0.05, Fig. 6C).

### Risk score is associated with lipid metabolism disorders and prognosis in HCC patients

**Figure 7:**
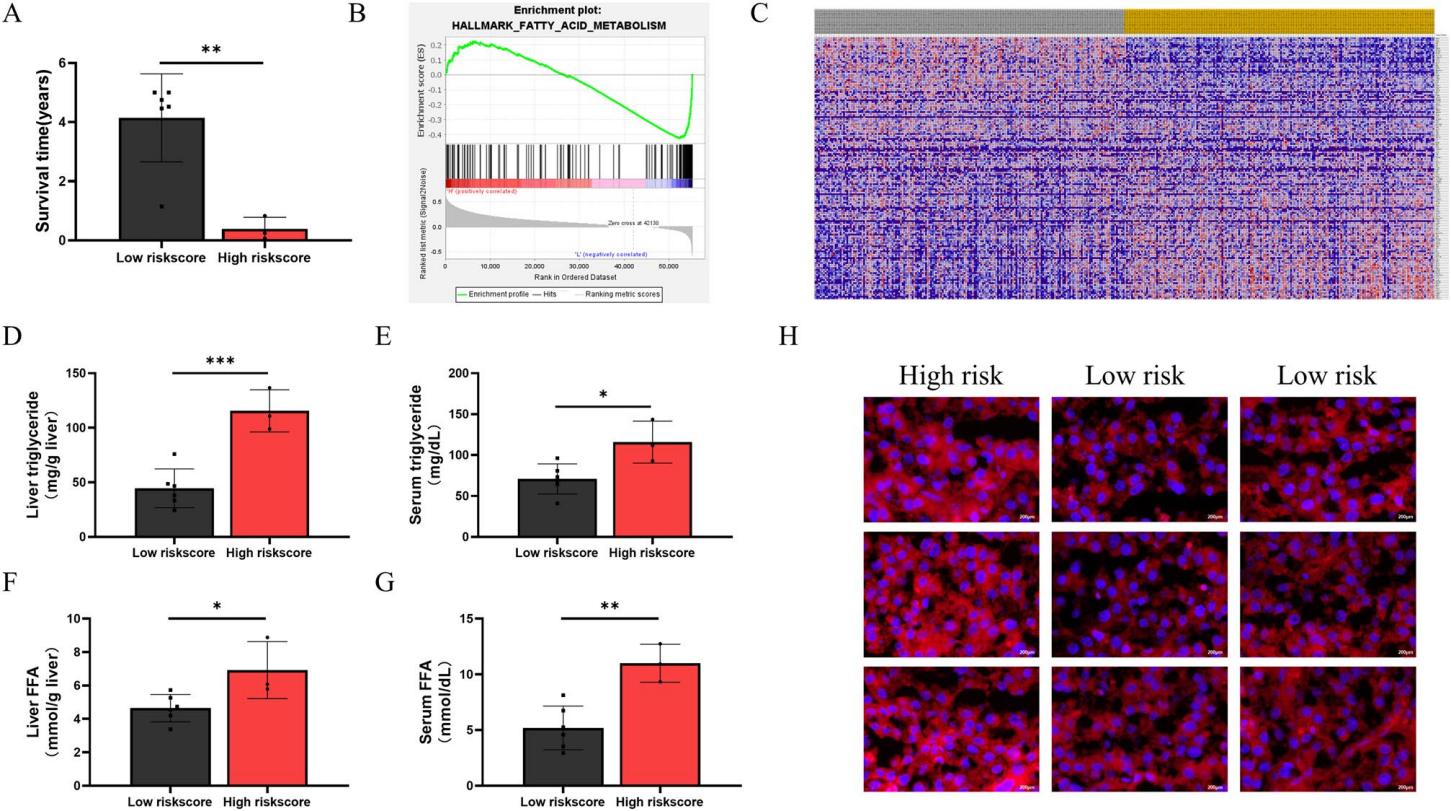
Risk score is associated with lipid metabolism disorders and prognosis in HCC patients. (**A**) Mean survival time between high-risk and low-risk groups (patients treated at People’s Hospital of Zhengzhou University). (**B**) Enrichment for the gene signature associated fatty acid metabolism between the low and high risk groups of TCGA dataset. (**C**) Heatmap (using “pheatmap” R package, https://cran.r-project.org/bin/windows/base/old/4.1.3/) showed differential expression of genes across all samples in TCGA-LIHC datasets. (**D**–**G**) TG and FAA levels in tumor tissue and serum of patients in high-risk and low-risk groups (patients treated at People’s Hospital of Zhengzhou University). (**H**) Nile red staining of tumor tissue from high-risk and low-risk patients (patients treated at People’s Hospital of Zhengzhou University (Scale bar, 200 μm).

According to the expression of six lipid metabolism-related genes, 40 tumor samples were divided into high and low expression groups based on each gene (High/Low = 1), respectively. Then, 3 HCC samples with high expression of APEX1, ME1, S100A10 and ACACA and low expression of ADH1C and CYP2C9 were taken as the high risk score group, and 6 HCC samples with low expression of APEX1, ME1, S100A10 and ACACA and high expression of ADH1C and CYP2C9 were selected as the low risk score group. Our results showed that the mean survival of HCC patients in the high-risk group was significantly lower in the lower-risk group (Fig. 7A). To further confirm the potential of risk scoring models in assessing lipid metabolism in HCC patients, GSEA analysis from the TCGA cohort revealed significantly enriched fatty acid metabolism genes coexpressed with either high or low risk scores samples (Fig. 7B). The heat map showed that compared with the low-risk group, the high-risk group was significantly enriched in lipid synthesis-related genes and was negatively correlated with lipid degradation-related genes (Fig. 7C). And the tumor tissue and serum triglyceride levels of HCC patients in the high-risk group were significantly increased, while the tumor tissue and serum FFA levels also showed the same trend (Fig. 7D–G). Nile red results from HCC patients showed that tumor samples from the high-risk group exhibited significant lipid accumulation compared to the low-risk group (Fig. 7H). The above results indicated that risk scores based on six lipid metabolism-related genes were significantly associated with lipid metabolism disorders in HCC.

### High risk score is associated with immunosuppression in HCC patients

**Figure 8:**
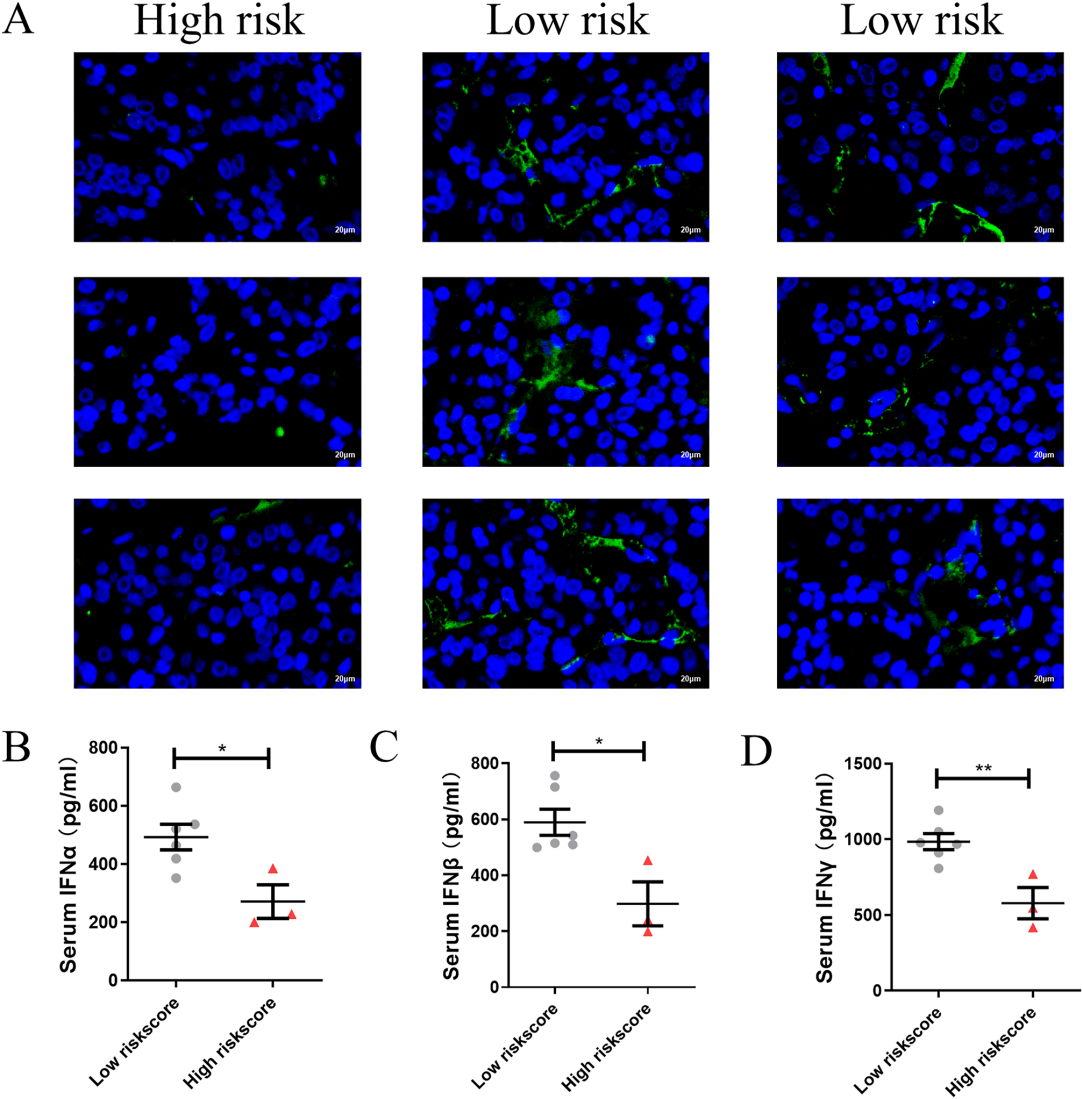
High risk score is associated with immunosuppression in HCC patients. (**A**) Representative images from multiplex immunofluorescent tissue staining for CD56 (green) and DAPI (blue) on high-riskscore and low-riskscore HCC tissues (Scale bar, 20 μm). (**B**–**D**) Serum IFNα, IFNβ and IFNγ concentration in high-riskscore and low-riskscore HCC patients.

The number of NK cells in HCC tissues and the concentration of IFN in serum were measured as part of our investigation into immune functions in high-risk patients. Our results showed that the infiltration of NK cells was significantly reduced in HCC samples from the high-risk group compared to the low-risk group (Fig. 8A). In addition, serum IFNα, IFNβ and IFNγ concentrations were also significantly lower in the high-risk group (Fig. 8B–D). The above result confirms the immunosuppressed status of high-riskscore patients.

## Discussion

One of the most common cancers and one of the ones with the highest morbidity and mortality rates is HCC^1^. A challenge of treating HCC is that existing therapeutic targets lack effectiveness or have a large limitation that makes them hard to apply to all patients. Another point is that the prognosis of patients is difficult to predict^2–4^. Therefore, providing clinical guidance to patients requires more effective targets and more accurate models. The evidence indicates that lipid metabolism disorders can influence a number of physiological and pathological processes, including tumorigenesis, development, metabolism, and tumor immunity^10,11^. However, the impact of lipid metabolism disorders on the prognosis of HCC remains to be elucidated. In addition, the current role of lipid metabolism in HCC still focuses on several key enzymes that are generally recognized, but the metabolism of tumors is complex. Therefore, more possible targets need to be studied to improve this vast regulatory network^12^. Using bioinformatics, we screened six genes for their potential to influence tumor lipid metabolism (ADH1C, APEX1, ME1, S100A10, ACACA, and CYP2C9), and a prognosis model for HCC patients was constructed.

We screened 94 differential genes related to lipid metabolism in HCC using limma package analysis (log foldchange > 2, *p* < 0.01). According to the survival analysis, 27 differential genes related to lipid metabolism related to prognosis were further screened out. Future research on the mechanism of action of these 27 genes is warranted. Based on LASSO regression analysis and multi-cox regression analysis, a prognostic model was developed using six lipid metabolism-related genes. A prognostic model can predict a patient's prognosis, and survival prediction and risk assessment can help in clinical management. Additionally, our model has been validated using two external databases, demonstrating its reliability.

The use of immunotherapy in cancer treatment has gradually increased over the past decade, and it has gradually become a research hotspot to investigate the role of cellular metabolic reprogramming in the tumor microenvironment on tumor immunity^13^. Disorders of lipid metabolism can regulate tumor growth by affecting immune cell infiltration, and lipid metabolism disorders regulate immune cell differentiation and function, thereby altering antitumor responses. Growing evidence suggests that altered energy metabolism may be the cause of antitumor immune failure, but few studies have evaluated the relationship between lipid metabolism-related genes and infiltration of immune cells^14,15^. Analyses of immune cell infiltration revealed that lipid metabolism-related genes were involved in the immune microenvironment and immune cell activity of the tumor. Three datasets all showed that risk scores are associated with inhibited IFN class I and class II response, and led to a decrease in NK cells (Fig. 5A–H, Figure S2A–H, Figure S3A–H). In addition, we confirmed this by immunofluorescence and section staining in HCC tissues from People’s Hospital of Zhengzhou University (Fig. 8A–D). According to reports, as well as recognizing and killing a target cell, NK cells also release cytokines to control antiviral immunity^16^. Additionally, NK cells produce antiviral cytokines and chemokines that link innate and adaptive immune responses^16^. Our results suggest that patients in the high-risk group have a reduced ability to kill tumor cells because of the antitumor effects of NK cells and IFN response, and may be caused by suppressed antiviral immunity, but the specific mechanisms underlying these relationships remain unclear. Therefore, it may be possible to uncover new immunotherapy targets by further studying the genes related to lipid metabolism we screened. Immunotherapy combined with lipid metabolism-related targets, such as adjuvant antiviral immunotherapy, may be a potential therapeutic approach.

Furthermore, few previous studies have linked lipid metabolism disturbances in HCC patients with their prognosis. Our results showed that patients in the high-risk score group had significantly lower survival than low-risk patients, and all three high-risk patients died within 1 year of onset (Fig. 7A). At the same time, high-risk patients had significantly higher levels of TG and FFA in tumor tissue and serum than low-risk patients (Fig. 7D–G). This was more intuitively confirmed by the Nile Red results of HCC samples (Fig. 7H). The above results indicate that these genes are very likely to regulate HCC lipid metabolism, resulting in its poor prognosis. In addition, many studies have demonstrated that lipid metabolism in cancer can regulate the immune microenvironment, and our results reveal possible targets for linking the two, which lays solid clinical evidence for future in-depth mechanism research.

A limitation of this study is the lack of RNA-sequencing data and follow-up data to validate the accuracy of our prediction model in predicting survival and classification of patients, as well as its assessment of lipid metabolism status in HCC, and its actual prediction performance still needs future large clinical samples to verify. But the constructed prognostic model proved to be useful for predicting patient survival, and the prediction performance has been validated in external ICGC datasets and GSE54236. Furthermore, targeting lipid metabolism in cancer cells is very promising since lipid metabolism disorders are rare in normal cells. Our predictive model can provide guidance for clinical work, drug therapy target selection and molecular marker research for HCC.

## Supplementary Information

Supplementary Figures.

Supplementary Tables.

## Data Availability

The data that support the findings of this study are available from the corresponding author upon reasonable request.

## Abbreviations

HCC: Hepatocellular carcinoma
TCGA-LIHC: The Cancer Genome Atlas-Liver Hepatocellular Carcinoma
TME: Tumor microenvironment
TIME: Tumor immune microenvironment
