# Supplementary material for: A six lipid metabolism related gene signature for predicting the prognosis of hepatocellular carcinoma
Source: Sci Rep. 2022 Dec 1;12:20781. doi: 10.1038/s41598-022-25356-2 (PMC9715694; doi:10.1038/s41598-022-25356-2)
Supplement: Supplementary file 1 — Supplementary Figures. [file 41598_2022_25356_MOESM1_ESM.pdf]

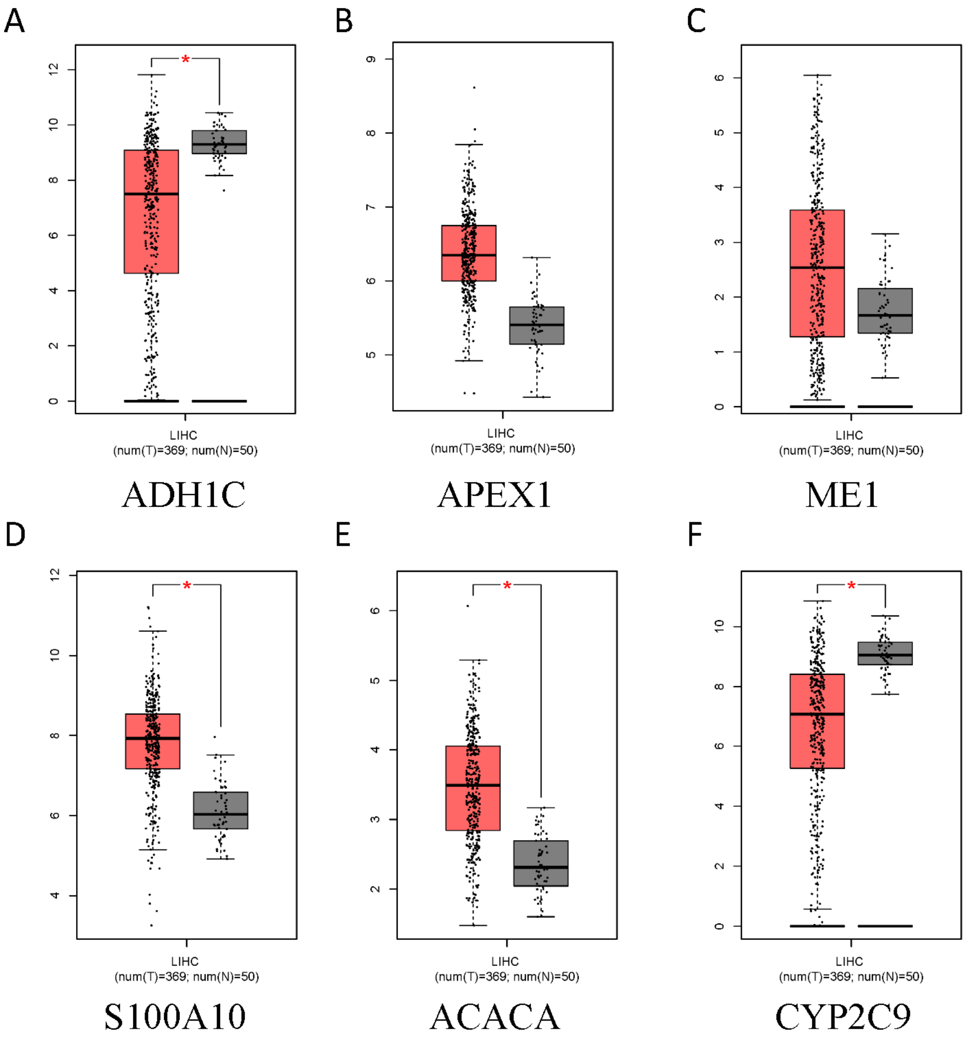

Figure S1

Gene expressions levels of the prognostic model. **A–F** Gene expressions levels of the prognostic model in TCGA.

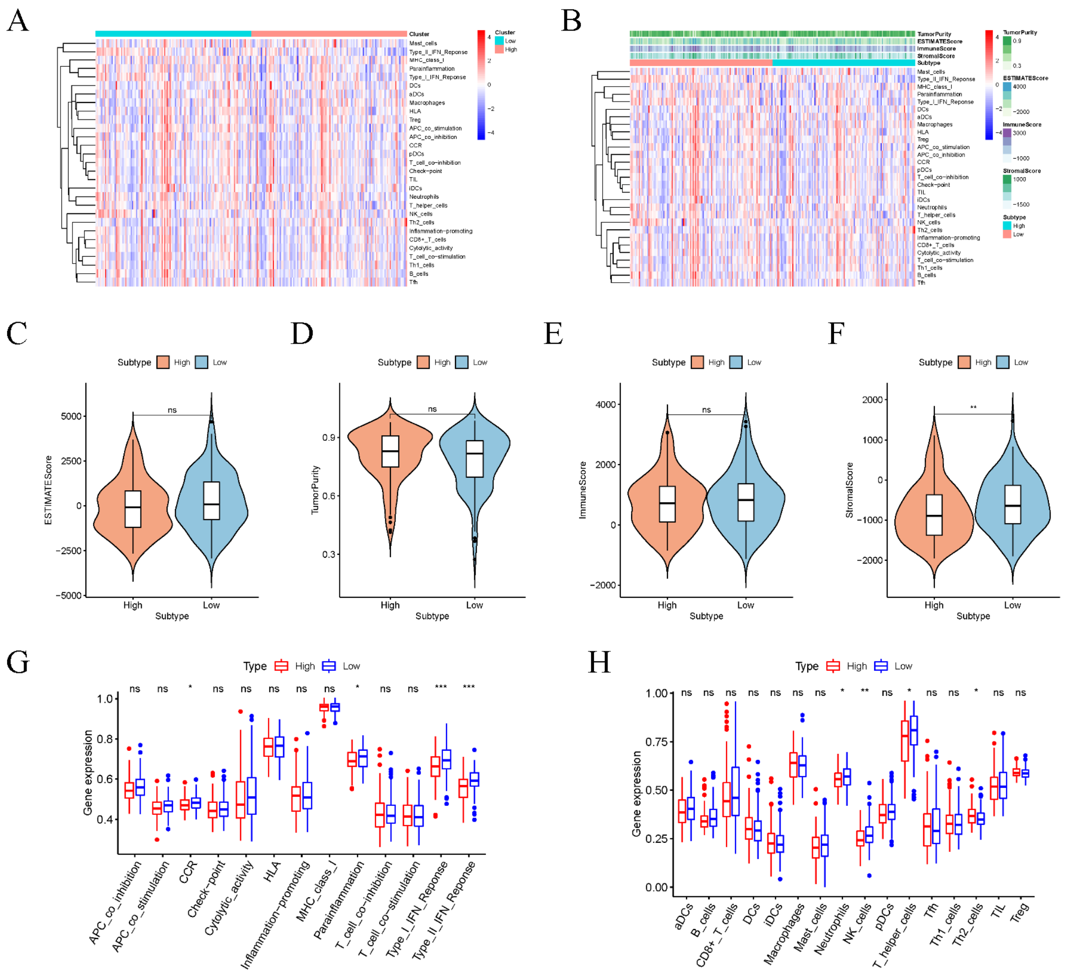

Figure S2

Risk score is closely related to tumor immune environment characteristics and immune infiltration in HCC. **A-B** Heat map (using " pheatmap" R package, https://cran.r-project.org/bin/windows/base/old/4.1.3/) distribution of risk score and various immune scores. **C–F** Comparison of estimate score, stromal score, immune score, and tumor purity between high-risk groups and low-risk groups. **G** The relationship between immune function and risk score in ICGC cohort. **H** The relationship between immune cell infiltration and risk score in the ICGC cohort.

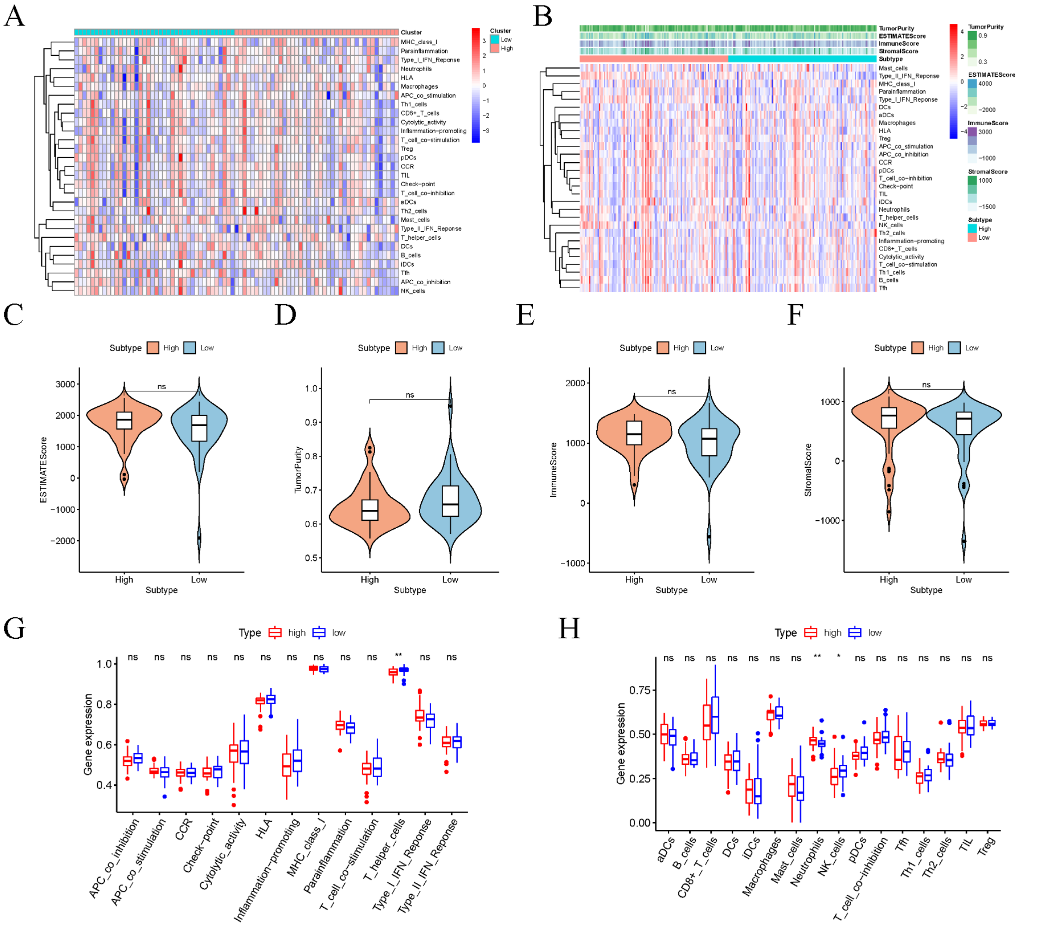

Figure S3

Risk score is closely related to tumor immune environment characteristics and immune infiltration in HCC. **A-B** Heat map (using " pheatmap" R package, https://cran.r-project.org/bin/windows/base/old/4.1.3/) distribution of risk score and various immune scores. **C–F** Comparison of estimate score, stromal score, immune score, and tumor purity between high-risk groups and low-risk groups. **G** The relationship between immune function and risk score in GSE54236 cohort. **H** The relationship between immune cell infiltration and risk score in the GSE54236 cohort.
