# Supplementary material for: A six lipid metabolism related gene signature for predicting the prognosis of hepatocellular carcinoma
Source: Sci Rep. 2022 Dec 1;12:20781. doi: 10.1038/s41598-022-25356-2 (PMC9715694; doi:10.1038/s41598-022-25356-2)
Supplement: Supplementary file 2 — Supplementary Tables. [file 41598_2022_25356_MOESM2_ESM.pdf]

Supplementary Table S1 Online analysis website

website	URL
TCGA-LIHC	https://portal.gdc.cancer.gov/
ICGC	https://dcc.icgc.org/
GSE54236	https://www.ncbi.nlm.nih.gov/geo/
GEPIA	http://gepia.cancer-pku.cn/
GSEA	https://www.gsea-msigdb.org/gsea/msigdb/index.jsp
TIMER	https://cistrome.shinyapps.io/timer/
HPA	https://www.proteinatlas.org

Supplementary Table S2 Primer sequences used in this study.

Primer name		Sequence (5’-3’)
ACC	F	ATGTCTGGCTTGCACCTAGTA
	R	CCCCAAAGCGAGTAACAAATTCT
APEX1	F	AGAGCCAGAGGCCAAGAAGAGTA
	R	GAAGCCCATCCACATTCCAAGAG
ADH1C	F	CCCCAAACTTGTGGCTGACTT
	R	CAGGACGGTACGGATACTCTTT
ME1	F	CTTCATGCCTGACTGATAT
	R	GACGTCTCAGGACTTACG
CYP2C9	F	GGGAAAGUAGAAGAGCAGATGGCCTG
	R	GGAGACAUGACTCAGCAAATAATAATGCTTT
S100A10	F	CACACCTTGATGCGTCCTCT
	R	GGCAACCGGATGCAAACAAT
β-Actin	F	ATCATGTTTGAGACCTTCAACA
	R	CATCTCTTGCTCGAAGTCCA
